# The Use of Virtual Therapy in Cardiac Rehabilitation of Female Patients with Heart Disease

**DOI:** 10.3390/medicina57080768

**Published:** 2021-07-28

**Authors:** Sandra Jóźwik, Błażej Cieślik, Robert Gajda, Joanna Szczepańska-Gieracha

**Affiliations:** 1 Faculty of Physiotherapy, University School of Physical Education in Wroclaw, 51-612 Wroclaw, Poland; j_sandra@wp.pl (S.J.); joanna.szczepanska@awf.wroc.pl (J.S.-G.); 2 Faculty of Health Sciences, Jan Dlugosz University in Czestochowa, 42-200 Czestochowa, Poland; 3 Center for Sports Cardiology at the Gajda-Med Medical Center in Pułtusk, 06-102 Pultusk, Poland; gajda@gajdamed.pl

**Keywords:** virtual reality, cardiovascular diseases, psychosomatic disorders, stress, cardiac rehabilitation, women

## Abstract

*Background and Objectives*: Cardiovascular disease (CVD) has become increasingly prevalent in women, and it is also in this group that the risk of developing depression is the highest. The most commonly applied therapeutic intervention in cardiac rehabilitation is Schultz’s autogenic training, which has proven to be of little efficacy in reducing depression and anxiety disorders. At the same time, a growing number of scientific reports have been looking at the use of virtual reality (VR) to treat mental health problems. This study aimed at assessing the efficacy of virtual therapy in reducing levels of depression, anxiety, and stress in female CVD patients. *Materials and Methods*: The study included 43 women who were randomly divided into two groups: experimental group (*N* = 17), where eight-week cardiac rehabilitation was enhanced with VR-based therapeutic sessions, and control group (*N* = 26), where the VR therapy was replaced with Schultz’s autogenic training. Mental state parameters were measured using the Perception of Stress Questionnaire and Hospital Anxiety and Depression Scale (HADS). *Results*: In the experimental group, the sole parameter which failed to improve was HADS-Anxiety, which remained at the baseline level. In the control group, there was a deterioration in nearly all tested parameters except for HADS-Depression. Statistically significant differences in the efficacy of rehabilitation were recorded in relation to the level of stress in the sub-scales: emotional tension (*p* = 0.005), external stress (*p* = 0.012), intrapsychic stress (*p* = 0.023) and the generalized stress scale (*p* = 0.004). *Conclusions*: VR therapy is an efficient and interesting complement to cardiac rehabilitation, with proven efficacy in reducing stress levels.

## 1. Introduction

Cardiovascular disease (CVD) constitutes a major health, social and economic problem worldwide. In Poland, the Central Statistical Office estimates that, at the current rate of morbidity and population aging, by 2050 the number of deaths due to CVD will increase to 209,900 annually [1]. Although until a dozen years ago heart disease was considered to be the domain of men, presently we know that CVD is actually prevalent in women. The mortality rate in women is 10% higher compared to men [1]. Risk factors for CVD, such as hypertension, diabetes, smoking, dyslipidemia and obesity are the same in men and women, but their influence on the frequency of morbidity seems to be gender-dependent [2]. The literature contains a growing number of studies on the aspect of gender in relation to the incidence of heart disease. Men have twice the incidence of coronary artery disease (12.3%) as women (6.4%). However, there seems to be higher morbidity in older women than in men [3]. Diseases such as angina pectoris, heart failure and stroke and the resulting complications leading to death are more common in women [4].

The Natpol 2011 study, modelled on the Framingham Heart Study and Nutrition Examination Survey (NHANES), determined the average heart age of a typical Polish person. The study demonstrated that cardiac aging occurs differently in men and women, as in men the process is gradual while in women it is sudden in nature. Women in their 30s have healthy hearts whose age is close to their biological age. The situation deteriorates rapidly after the age of 40, as then women’s hearts are 10 years older than their metric age. When women reach the age of 60, the condition of their heart worsens dramatically and the heart of a 70-year-old Polish woman is 95 years old [5]. Consequently, in Poland, women have higher morbidity from CVD but with the onset occurring 10–15 years later than in men [6].

One must not disregard psychosocial factors with significant impact on the risk of cardiac events, such as depression, anxiety disorders, or low social support. In childhood and early adolescence, there is no gender-specific difference in the epidemiology of depressive disorders [4]. The literature increasingly considers hormonal transition periods, i.e., the differences occurring in mid-adolescence and in menopause, as important moments with an impact on the occurrence of depression in women. In his study, Steiner et al. describe elevated rates of depression during the perinatal and peri-menopausal periods, suggesting the influence of steroid hormones (17-β-estradiol and progesterone) [7]. Estrogen has very important functions in the female body, as it inhibits the development of atherosclerosis and prevents vascular wall remodeling and blood clots. It also lowers blood pressure and glucose levels, thus preventing diabetes. In a situation where estrogen levels begin to decline dramatically, the condition of the heart and blood vessels may rapidly deteriorate. Women after menopause no longer benefit from the cardioprotective effects of estrogen and thus are at an increased risk of CVD [8]. 

Recently, virtual reality (VR) has been increasingly used as a tool supporting the therapy of psychological and psychiatric diseases. In 2020, Cieślik et al. in their systematic review of reviews, stated that an impact on stress and anxiety were among the most frequently studied effects of the application of VR and confirmed the positive impact of VR therapy in all reviews analyzed (23 reviews, 12,991 individuals in total) [9]. VR has even found applications in reducing fear of flying and fear of heights. In their study, Wallach et al. described an effective application of VR solutions in the treatment of flying phobias [10], while Hong et al. found that a self-training VR program aimed at alleviating the fear of heights may be safely used in a home or hospital setting, allowing the user to stop practicing at any time or returning to a specific anxiety-inducing situation [11]. In fact, the efficacy of a therapeutic technique using VR was examined in the treatment of acrophobia in students as early as in 1995, with a resulting significant reduction in symptoms of the treated condition [12]. In their review, Jerdan et al. confirmed the beneficial impact of VR in reducing symptoms of anxiety and its efficacy in treating pain [13]. Zeng et al., Lee et al. and Maples-Keller et al. all concluded that the use of VR has a beneficial effect on reducing anxiety symptoms; furthermore, no studies have reported any harmful effects related to the use of this modern technology. Research conducted by the above authors provides evidence to confirm the wealth of potentially related to the use of VR in treating anxiety disorders [14,15,16]. 

One of the major goals of cardiac rehabilitation (CR) is an improvement of the patient’s quality of life. Although we know how important mental health is for cardiac patients, depressive and anxiety disorders are still under-recognized and undertreated, especially in women [17]. Therefore, the aim of this study was to assess the influence of VR therapy on the reduction of anxiety and improvement of mood in women undergoing CR.

## 2. Materials and Methods

### 2.1. Participants

The study encompassed 52 women who were randomly divided into two groups (experimental and control). In the experimental group, nine participants did not complete the study due to religious beliefs, vision and hearing problems, pacemaker concerns, or organizational problems (the VR therapy schedule conflicted with the patients’ home responsibilities). Finally, 43 women participated in the study, with 17 subjects in the experimental group and 26 in the control group. The mean age was 65.4 (standard deviation, SD 8.0) years. Inclusion criteria were diagnosed ischemic heart disease and female gender. Exclusion criteria were withdrawal of patient consent at any stage of the study, male gender, cognitive impairment preventing self-completion of study questionnaires, presence of consciousness disorders, psychotic symptoms, bipolar affective disorder or any other serious psychiatric disorders, initiation of psychiatric or psychological treatment during the course of the study. Also, contraindications for virtual therapy included epilepsy or vertigo.

The participants were recruited from the PRO CORDE Cardiology Center in Wroclaw, Poland. All subjects voluntarily entered the study, which was confirmed with their written consent. They were thoroughly informed of the course of the study and that they could discontinue participation at any time without consequences. The study was carried out in accordance with the guidelines of the Declaration of Helsinki and was approved by the bioethics committee (No. 31/2019) in July 2019.

### 2.2. Procedures

All the participants were undergoing standard CR of equal duration for each patient, namely eight weeks. In the course of rehabilitation, they took part in three exercise sessions per week, 1 h 45 min per session. The exercise intensity level was determined individually for each subject and ranged from 60% to 85% of the maximum heart rate. Prior to the commencement of exercises, participants underwent an echocardiographic stress test, which was repeated at the end of rehabilitation. Before the beginning of each session, blood pressure measurements were taken in each patient, to be later repeated at the end of the session. Exercise sessions began with cycle-ergometer exercises and took 40 min. Blood pressure was taken during the highest workload. A physiotherapist monitored the patients’ echocardiographic measurements during the entire cycle-ergometer workout. After completing the workout, the patients participated interchangeably in medical general fitness training or in cardio fitness training, with both types of training lasting for 40 min. The cardio fitness training used a treadmill, a rowing machine, a multi-fitness station, an elliptical machine and a cycle ergometer. The subjects exercised for 5 min using each machine, with a 2-min break in between. Blood pressure measurements were taken after training, and the subjects’ heart rate was monitored during the exercises.

In the control group (*N* = 26), patients participated in standard CR complemented with eight sessions of Schultz’s autogenic training. The sessions were led by a psychologist, and the patients lay on mattresses while relaxing. Relaxation consisted of six basic exercises, i.e., inducing feelings of heaviness (e.g., heavy hands) and warmth (warm hands), heart regulation (calm heart), and breathing (calm breathing). Then, the feeling of warmth was induced again, but this time in the solar plexus (warm body), to be finally followed with the feeling of a cool forehead. The relaxation sessions were played from a compact disk (CD). The goals of the therapy were: general calming and relaxation, alleviating the feelings of anxiety, sadness, depression and fear, as well as supporting the treatment of depression and sleep problems.

Patients in the experimental group (*N* = 17) also took part in the standard CR, but instead of Schultz’s autogenic training, they received eight therapeutic sessions using the VR TierOne device (Stolgraf^®^, Stanowice, Poland). The VR set comprised a computer dedicated to processing 3D graphics, VR goggles displaying high-resolution images with great picture quality (90 Hz) and manipulators transferring the rehabilitee’s hand movements into the VR world. The computer provided sufficient computing power for the real-time transfer of the user’s movements into the virtual environment. The entire experience was made whole by surround sound effects.

The idea of the therapy is based on the metaphor of the Virtual Therapeutic Garden, where the patient could calm down and relax while performing the task of coloring therapeutic mandalas that changed with every session. The VR TierOne provided the patients with a multisensory experience (involving sight, hearing and kinesthetics), which intensified the process of immersion in the virtual world. The patient was visually rewarded for completing her tasks as the garden became more beautiful and vibrant with each session. This was a metaphor related to the process of treatment and rehabilitation. The goals of the therapy were calming and putting the patient in a state of psychophysical relaxation, recalling associations related to previous pleasant sensations, improving mood, reducing the level of anxiety, increasing motivation for active participation in the rehabilitation process, cognitive activation and stimulation of the patient’s creativity.

The content of the VR therapy was developed by Joanna Szczepańska-Gieracha, a certified European Association of Psychotherapy (EAP) under the supervision of Krzysztof Klajs, chairman of the Scientific Department of Psychotherapy of the Polish Psychiatric Association (PPA). The VR TierOne medical device was developed under a grant received from the National Centre of Research and Development (NCRD). The therapeutic method was described in earlier publications [18,19,20,21].

### 2.3. Outcome Measures

Baseline assessments were carried out in both groups at the start of CR and at the final assessment three weeks into rehabilitation. Two standardized psychometric tools (the Perception of Stress Questionnaire, PSQ and the Hospital Anxiety and Depression Scale, HADS) and a self-developed questionnaire were used to collect sociodemographic and clinical data, as well as data related to the lifestyle of the participants.

The PSQ was used to measure the participants’ perceived levels of stress, including generalized stress levels, emotional tension, external and intrapsychic stress. The questionnaire comprises 27 statements, with 21 referring to the individual components of stress and six referring to the Lie Scale. The respondent determined the degree to which a given statement concerned her, using a five-point Likert scale. The higher the score, the more severe the symptoms of stress [22].

Another research tool was the HADS, used to assess the level of depression and anxiety disorders. The HADS is a 14-item questionnaire scored from 0 to 3 where seven items relate to anxiety (HADS-A), while the remaining seven relate to depression (HADS-D). The global scoring ranges from 0 to 42, with a cut-off point of 8/21 for anxiety and 8/21 for depression. The higher the score, the greater the intensity of anxiety or depression [23].

The last research tool was a self-developed questionnaire which served to collect sociodemographic and clinical data, as well as data related to the participants’ lifestyle. The questionnaire included questions regarding the following conditions: myocardial infarction (MI) classified as either an ST segment elevation MI (STEMI) or a non-ST segment elevation MI (NSTEMI), paroxysmal atrial fibrillation, Takotsubo syndrome (broken heart syndrome), and cardiomyopathy. It also collected data regarding the received procedures, such as an implanted pacemaker, heart stimulator, percutaneous coronary intervention (PCI), coronary artery bypass grafting (CABG), electrode replacement or artificial valve implantation. The collected data had confirmation in the patients’ medical records. The questionnaire also contained questions related to respondents’ level of education, marital status and employment, presence of diabetes (Table 1).

### 2.4. Data Analysis

All analyses were performed using STATISTICA 12 software (StatSoft, Palo Alto, CA, USA). The chi-squared test was used to assess significant relationships between variables. Comparisons between the two groups (at baseline and final measurement points) were carried out using Student’s t-test for independent trials. The significance level was set at *α* < 0.05.

## 3. Results

### 3.1. Participants Characteristics

All women had been diagnosed with ischemic heart disease. PCI had been performed in 35% and CABG in 7% of the participants. MI had been diagnosed in 35% of the women, of whom 14% had STEMI and 21% NSTEMI. An implanted device (pacemaker or stimulator) was reported by 10% of the participants. The procedure of artificial valve implantation concerned 5% and electrode replacement 2% of the participants. The rest of the subjects had been diagnosed with paroxysmal atrial fibrillation, cardiomyopathies and Takotsubo syndrome (2%) (Table 1). Diabetes had been diagnosed in 26% of the respondents. The mean body mass index (BMI) score in the studied group of women was 27.3, and in 65% of the subjects, the score was ≥25, indicating overweight. A higher level of education was reported by 42% of the respondents; a slightly smaller proportion of 30% had secondary education and 28% of the women had primary/vocational education. Most participants were married (47%) or widowed (30%), 16% were single, and 7% were divorced. Of the recruited group, 74% were retired and 14% were actively employed, 7% were on a disability pension and 5% were unemployed. As far as stress coping ability was concerned, 49% were of the opinion that they had that ability, while 21% stated that they lacked stress-coping skills. Of the survey respondents, 37% observed that the presenting illness affected their relationship with others; the remaining 63% did not notice a difference in this respect. A very high proportion of respondents experienced sleep problems, with 77% reporting occasional or frequent insomnia and 12% reporting excessive sleepiness. In the opinion of 56% of respondents, their health was in poor condition; 28% assessed their health as average and only 16% of women stated that they were in good health. Of the women surveyed, 67% said that they feared for their lives in the context of a past illness or cardiac procedure (Table 1). No statistically significant differences regarding the type and number of diagnosed conditions were found between the experimental and control group. It was shown that the distribution of the analyzed characteristics did not differ between the experimental and control group (*p* < 0.05).

### 3.2. Results for the Mental State in the Studied Groups

**Figure 1:**
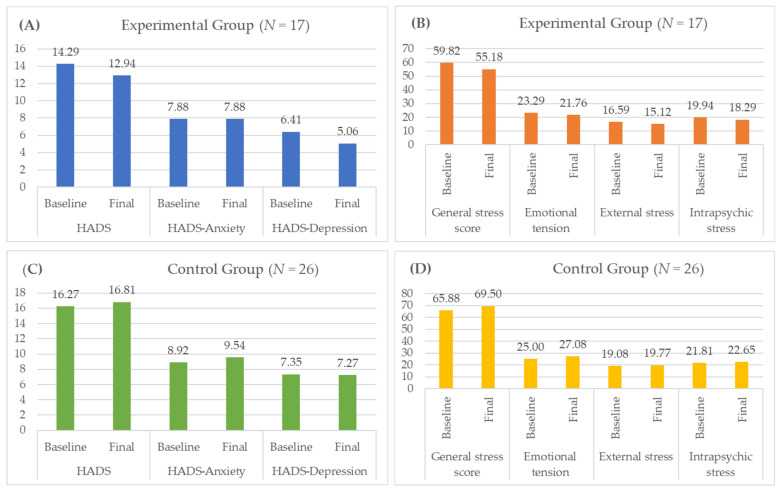
Mean values in the baseline and final measurement for Hospital Anxiety and Depression Scale (HADS) and stress level for Experimental (**A**,**B**) and Control (**C**,**D**) groups.

In the mental status survey conducted in the experimental group, most of the parameters studied improved significantly: HADS (14.29 vs. 12.94), HADS-D (6.41 vs. 5.06), emotional tension (23.29 vs. 21.76), external stress (16.59 vs. 15.12), intrapsychic stress (19.94 vs. 18.29) and the generalized stress scale (59.82 vs. 55.18). The sole parameter in the experimental group that did not improve was HADS-A (7.88 vs. 7.88) (Table 2, Figure 1).

In contrast, in the control group, most of the assessed mental state deteriorated: HADS-A (8.92 vs. 9.54), HADS (16.27 vs. 16.81), emotional tension (25.00 vs. 27.08), external stress (19.08 vs. 19.77), intrapsychic stress (21.81 vs. 22.65) and the generalized stress scale (65.88 vs. 69.50). The only parameter that deteriorated only slightly was HADS-D (7.35 vs. 7.27) (Table 2, Figure 1).

Statistically significant differences in the efficacy of rehabilitation between groups were recorded in relation to the level of perceived stress in the sub-scales: emotional tension (*p* = 0.005), external stress (*p* = 0.012), intrapsychic stress (*p* = 0.023) and the generalized stress scale (*p* = 0.004).

## 4. Discussion

Our study was carried out in a group of women, as women are most at risk of experiencing depression and anxiety. It has been known for a long time that depression and anxiety disorders are independent predictors of the incidence of CVD and often lead to increased mortality [24,25,26,27,28,29]. In their studies, Ladwig, Barnes and Albus pointed to the occurrence of mental disorders, in particular depression, anxiety and cognitive disorders in the course of coronary artery disease, especially in women [30,31,32]. The choice of the study group and the nature of the problem of depression and anxiety disorders which was addressed in our study also had its foundation in research by Piepenburg, who confirmed the occurrence of depression in heart disease, focusing on heart failure and noting that depression affects women more often than men [33].

Among the emerging technologies that can contribute to the treatment of anxiety, depression and stress disorders, VR appears to be the most exciting and advanced [14,34]. Increasingly more studies confirm the efficacy of VR therapies in fields such as psychology and psychiatry [9,35,36]. Our results also confirm the efficacy of the applied VR-based therapy in reducing the symptoms of stress. In their study, Jerdan et al. concluded that VR is a promising form of relaxation [13]. However, most studies on the use of VR are related to the treatment of anxiety disorders, including arachnophobia [36,37,38,39] or social phobia, where Gebara et al. described improvement in the area of a social anxiety disorder (SAD) across all scales used, and their study showed that exposure to VR leads to better treatment adherence and a reduction in SAD symptoms [40]. In their study, Urech et al. observed an improvement in SAD symptoms as compared to pre-study and follow-up assessment and suggested that VR is a feasible and promising therapy medium [35]. Another study described the use of VR to treat the symptoms of SAD involving techniques characteristic of cognitive-behavioral therapy (CBT). The VR intervention helped reduce the symptoms of SAD. The authors concluded that using VR can be more advantageous over standard CBT and can constitute a practical solution for therapists [41].

However, in our study, the only parameter that failed to improve in the VR group was HADS-Anxiety. On the other hand, no increase in the intensity of anxiety symptoms was observed, contrary to what was found in the control group. We may surmise that it was thanks to the applied VR therapy that anxiety remained at the baseline level and did not deteriorate as it did in the control group. We can agree with the above authors that VR not only has the potential to reduce anxiety symptoms, but it also appears to be a safe method [14,16], as we did not note any harmful effects or worsening of anxiety symptoms in the experimental group. We found no studies in the available literature that would concern the use of VR in treating anxiety disorders in a group of women with CVD undergoing CR. Not being able to refer to other studies makes it difficult for us to identify the factor responsible for anxiety being the only parameter that failed to improve. We acknowledge the need for further research relating to the use of VR in treating anxiety conducted in a larger group of female cardiac patients.

Considering the high incidence of mood disorders in the general population, it seems that the problem of depression is still too rarely analyzed in studies involving VR interventions [9,13]. In their review, Jerdan et al. described a successful application of VR in treating depression. Patients demonstrated a marked reduction in depression severity and self-criticism, with a substantial increase in self-compassion and self-acceptance [13]. In their literature overview, Park et al. mentioned a study carried out at the University of Barcelona (Spain) and involving the application of VR therapy, where the intervention resulted in reduced severity of depressive symptoms and reduced self-degradation as well as an increased sense of satisfaction in the study’s participants [42].

The use of VR may also facilitate mindfulness practice by limiting distractions and increasing the sense of presence [42]. Lindner et al. described in their study attempts that were made to translate CBT techniques for depression into VR modality, including experiences such as psychoeducation, behavioral activation and cognitive restructuring. The authors describe the applied low-intensity, automated VR as an alternative for the existing CBT techniques that is ideal for treating depression and, thanks to the low cost of intervention (which does not involve a therapist), is an affordable and accessible consumer technology and constitutes a promising therapeutic solution that could have a huge impact on public mental health [43]. Migoya-Borja et al. examined 28 people with depressive disorders, 13 of whom had major depressive disorder to evaluate the new VRight software. They observed that VR-based interventions used as a psychoeducation tool may improve symptoms awareness in patients with depressive disorders. The VR-based software was well-accepted among depressive patients, and showed high levels of satisfaction [44].

The surge in the incidence of mental disorders, including an increase in depression levels caused by the COVID-19 pandemic, has led to a change in the way we think about this form of treatment and the need to open up to new, safe technologies. The above problem was recognized by Paul et al., who in their case report proposed the use of remote therapy as a replacement method for CBT. The patient in the study participated for four weeks in a weekly psychotherapy Zoom session, conducted in the home setting. The authors emphasized that the aim of the study was to test the feasibility, acceptability and tolerability of this form of therapy delivery in patients with depression. A significant improvement in the severity of symptoms was achieved [45].

In our study, we obtained a reduction in depressive symptoms in the experimental group (6.41 vs. 5.06) as compared to the control group (7.35 vs. 7.27). The obtained value *p* = 0.066 encourages further research on the use of VR in CR. The literature contains studies confirming that depressive disorders are associated with increased activation of the sympathetic nervous system and decreased activation of the parasympathetic nervous system [4], and as we know, the work of the autonomic nervous system is significantly related to the level of perceived stress. In the present study, the best results were observed specifically in the reduction of stress symptoms. Results obtained in the experimental group were significantly better in all the assessed stress parameters compared to the control group. Bearing in mind that stress is a strong predictor of CVD, the obtained results may be of significant clinical relevance.

In their study, Guillén et al. compared the application of standard CBT protocols delivered following a traditional format to treatments involving a VR system “EMMA’s WORLD” in the therapy of stress-related disorders. The VR group obtained better results compared to the group receiving traditional treatments. Also, the VR system was well-received by patients and it helped foster motivation for further therapy [46]. Lognoul et al. described a novel approach in treating stress based on tolerating fear and not on replacing it. The authors noted that VR fits in well with the new approach as it allows emotional involvement from patients and represents a complementary approach to the classical modalities of exposure therapy [47]. In their study, Maarsingh et al. examined whether VR with real-time biofeedback would help in training people to develop a new mindset related to stress. They concluded that using a VR application may be useful in working toward a more positive stress mindset [48].

The current situation related to the COVID-19 pandemic and the barriers imposed in the form of physical distance, social isolation and quarantine as well as the emerging economic problems and uncertainty related to the future have aggravated the already existing problem of the prevalence of anxiety, depression and stress. This problem greatly affects cardiac patients because mental disorders are a predictor of the incidence of CVD, confirmed Goldstein et al., who reported that people with depression and anxiety disorder are more likely to develop acute MI, heart failure and are at increased risk of mortality [49]. Concurrently, a growing number of authors have noted that standard procedures for treating depressive-anxiety disorders in people with CVD have little efficacy [42,43,45,50,51], that CR is focused only on improving cardiorespiratory function, and psychological issues are continually marginalized. As early as 2012, Szczepańska-Gieracha et al. wrote that CR is not an effective treatment for depression and anxiety disorders [52]. Also in our study, we observed that standard CR only minimally reduced depressive symptoms (7.35 vs. 7.27), while the other parameters (anxiety and stress) even deteriorated in its course. The results obtained in the control group are a clear indication that CR alone did nothing to improve the mental state of the studied women.

The obtained outcomes show the necessity of making significant changes in the CR program, consisting of the introduction of modern, attractive and, above all, efficacious treatment methods. VR-based therapy may be an interesting complement to standard CR. In their study, Rizzo et al. observed that the use of VR leads to increased participation in rehabilitation, with patients reporting greater motivation to engage in the course of treatment, as the form of treatment offered appears more attractive compared with traditional rehabilitation approaches [53].

### Limitations

The present study has a number of significant limitations, such as a small population size and the lack of follow-up measurements to assess the long-term effects of the intervention used. In addition, a relatively large number of participants in the experimental group dropped out of the study after the first session due to religious reasons (reluctance to use any form of psychotherapy), visual problems (visual impairment making it difficult to use the VR goggles or past cataract procedure) or fear that the VR device might interfere with their pacemaker function. Therefore, the number of dropouts should be monitored in further studies. These limitations lead to a cautious interpretation of the presented results.

## 5. Conclusions

Our study demonstrated that virtual therapy is an effective method supporting cardiac rehabilitation in the reduction of stress symptoms in CVD patients. Statistically significant differences in the efficacy of rehabilitation between groups were recorded in relation to the level of perceived stress in the sub-scales: emotional tension, external stress, intrapsychic stress and the generalized stress scale. The therapy conducted in the Virtual Therapeutic Garden is an interesting alternative to Schultz’s Autogenic Training, but as a large number of recruited patients withdrew from the study due to concerns related to VR technology, the subject requires further research.

## Figures and Tables

**Table 1:** Participant baseline characteristics.

Variable	Total	Experimental Group	Control Group	*p*
*N*	43	17	26	-
Age, years (*SD*)	65.40 (7.98)	65.65 (10.07)	65.23 (6.49)	0.87
Body mass, kg (*SD*)	70.98 (12.76)	71.06 (13.26)	70.92 (12.68)	0.97
Height, cm (*SD*)	161.16 (6.09)	159.24 (4.72)	162.42 (6.63)	0.09
BMI, kg/cm^2^ (*SD*)	27.27 (4.41)	27.91 (4.46)	26.85 (4.40)	0.44
**Specific diagnosis, *N* (%)**				
Pacemaker	2 (4.65)	2 (4.65)	0	0.15
PCI	15 (34.88)	7 (16.28)	8 (18.60)	
STEMI	6 (13.95)	1 (2.32)	5 (11.63)	
NSTEMI	9 (20.93)	1 (2.32)	8 (18.60)	
CABG	3 (6.98)	2 (4.65)	1 (2.32)	
Heart stimulator	2 (4.65)	2 (4.65)	0	
Replaced electrode	1 (2.32)	1 (2.32)	0	
Cardiomyopathy	1 (2.32)	1 (2.32)	0	
Artificial valve implantation	2 (4.65)	0	2 (4.65)	
Takotsubo syndrome	1 (2.32)	0	1 (2.32)	
Paroxysmal atrial fibrillation	1 (2.32)	0	1 (2.32)	
Diabetes, *N* (%)	11(25.58)	4 (9.30)	7 (16.28)	0.80
**Education, *N* (%)**				0.31
Primary/vocational	12 (27.90)	5(11.63)	7 (16.28)	
Secondary	13 (30.23)	3 (6.98)	10 (23.25)	
Higher	18 (41.86)	9 (20.93)	9 (20.93)	
**Marital status, *N* (%)**				0.14
Married	20 (46.51)	7 (16.28)	13 (30.23)	
Single	7 (16.28)	5 (11.63)	2 (4.65)	
Divorced	3 (6.98)	2 (4.65)	1 (2.32)	
Widowed	13 (30.23)	3 (6.98)	10 (23.25)	
**Employment status, *N* (%)**				0.22
Employed	6 (13.95)	1 (2.32)	5 (11.63)	
Disability pension	3 (6.98)	1 (2.32)	2 (4.65)	
Retired	32 (74.42)	13 (30.23)	19 (44.19)	
Unemployed	2 (4.65)	2 (4.65)	0	

BMI—Body Mass Index; SD—Standard Deviation; PCI—percutaneous coronary intervention; STEMI—ST segment elevation myocardial infarction; NSTEMI—non-ST segment elevation myocardial infarction; CABG—coronary artery bypass grafting.

**Table 2:** Comparison of mental status in the experimental and control group before and after rehabilitation.

Characteristic	Measurement	Group		*p*
		Experimental (*N* = 17)	Control (*N* = 26)	
		Mean (SD)	Mean (SD)	
HADS	Baseline	14.29 (8.04)	16.27 (7.52)	0.42
	Final	12.94 (7.08)	16.81 (7.64)	0.10
HADS-Anxiety	Baseline	7.88 (4.27)	8.92 (4.21)	0.44
	Final	7.88 (3.69)	9.54 (4.17)	0.19
HADS-Depression	Baseline	6.41 (4.21)	7.35 (3.80)	0.46
	Final	5.06 (3.88)	7.27 (4.00)	0.07
General stress score	Baseline	59.82 (20.00)	65.88 (16.69)	0.29
	Final	55.18 (16.02)	69.50 (14.30)	0.004
Emotional tension	Baseline	23.29 (7.92)	25.00 (6.39)	0.28
	Final	21.76 (6.54)	27.08 (5.63)	0.005
External stress	Baseline	16.59 (6.21)	19.08 (6.15)	0.20
	Final	15.12 (5.87)	19.77 (5.53)	0.01
Intrapsychic stress	Baseline	19.94 (8.17)	21.81 (6.12)	0.40
	Final	18.29 (6.72)	22.65 (5.30)	0.02

HADS—Hospital Anxiety and Depression Scale; SD—Standard Deviation.

## Data Availability

Data is available from the corresponding author on a reasonable request.
